# microRNA-148a inhibits hepatocellular carcinoma cell invasion by targeting sphingosine-1-phosphate receptor 1

**DOI:** 10.3892/etm.2014.2137

**Published:** 2014-12-16

**Authors:** SHU-LIANG ZHANG, LING LIU

**Affiliations:** 1 Department of Hepatobiliary Surgery, Linzi District People’s Hospital, Zibo, Shandong 255400, P.R. China; 2 National Hepatobiliary and Enteric Surgery Research Center, Xiangya Hospital, Central South University, Changsha, Hunan 410008, P.R. China

**Keywords:** hepatocellular carcinoma, microRNA-148a, sphingosine-1-phosphate receptor 1, invasion

## Abstract

microRNA (miR)-148a has been shown to act as an important suppressor in numerous human malignancies and is markedly downregulated in hepatocellular carcinoma; however, the role of miR-148a in the regulation of hepatocellular carcinoma cell invasion, as well as the underlying mechanism, has never been studied. In the present study, the expression level of miR-148a was found to be significantly decreased in hepatocellular carcinoma tissues and HepG2 cells when compared with that in the normal adjacent tissues. Furthermore, a novel target of miR-148a was found, sphingosine-1-phosphate receptor 1 (S1PR1), whose expression was negatively regulated by miR-148a at a post-transcriptional level in hepatocellular carcinoma HepG2 cells. Upregulation of miR-148a by transfection with miR-148a mimics notably suppressed HepG2 cell invasion, similar to the effect of the SIPR1 downregulation induced by SIPR1-specific small interfering RNA, while the restoration of S1PR1 expression reversed the inhibitory effect of miR-148a upregulation on HepG2 cell invasion. Accordingly, the current study suggests that miR-148a plays an inhibitory role in the regulation of hepatocellular carcinoma cell invasion by directly targeting S1PR1.

## Introduction

Hepatocellular carcinoma is the third leading cause of mortality from cancer, with an increasing incidence worldwide. As a result, it is one of the most serious threats to health in the global population (1). Deregulation of oncogenes or tumor suppressors has been shown to be closely associated with the development and progression of hepatocellular carcinoma (2,3). Accordingly, the development of effective therapeutic targets for hepatocellular carcinoma is urgently required.

microRNAs (miRNAs) are non-coding RNAs formed of 18–25 nucleotides that can cause the inhibition of gene expression at a post-transcriptional level by directly binding to the 3′-untranslational region (UTR) of mRNAs (4). Deregulation of miRNAs, such as miR-204, miR-331, miR-125b, miR-148b and miR-148a, has been demonstrated to play an important role in hepatocellular carcinoma (5–9). Among these miRNAs, miR-148a has been demonstrated to act as a tumor suppressor in several types of cancer, including gastric, non-small cell lung and colorectal cancer (10–12). For instance, the expression level of miR-148a was reduced in gastric cancer tissues and cell lines, and it could regulate various target genes and pathways involving tumor proliferation, invasion and metastasis (10).

Deregulation of miR-148a has additionally been shown to affect the poor prognosis of hepatocellular carcinoma, associated with the overexpression of ubiquitin-specific protease 4, an identified target of miR-148a (9). miR-148a has also been found to suppress the epithelial-mesenchymal transition and metastasis of hepatocellular carcinoma cells by targeting Met/Snail signaling (13,14). As one miRNA can directly bind to numerous target mRNAs, whether other target genes exist in hepatocellular carcinoma remains unclear.

The present study aimed to explore the role of miR-148a in the regulation of hepatocellular carcinoma cell invasion. Furthermore, it was determined whether sphingosine-1-phosphate receptor 1 (S1PR1) acted as a downstream effector of miR-148a in hepatocellular carcinoma cells.

## Materials and methods

### Tissue specimen collection

This study was approved by the Ethics Committee of Shandong University (Jinan, China). Informed consent was obtained from the patients. Twenty hepatocellular carcinoma tissues and their matched adjacent normal tissues were collected in the Department of General Surgery, Qilu Hospital of Shandong University (Jinan, China). Tissue samples were immediately frozen in liquid nitrogen following surgical removal.

### Cell culture

Human hepatocellular carcinoma HepG2 cells were obtained from the Cell Bank of Shandong University and cultured in Dulbecco’s modified Eagle’s medium (Life Technologies, Carlsbad, CA, USA) with 10% fetal bovine serum (FBS; Pierce Chemical, Rockford, IL, USA) at 37°C in a humidified incubator containing 5% CO_2_.

### Reverse transcription-quantitative polymerase chain reaction (RT-qPCR) assay

Total RNA was extracted using TRIzol^®^ reagent (Invitrogen Life Technologies, Carlsbad, CA, USA). An miRNA Reverse Transcription kit (Invitrogen Life Technologies) was used to convert RNA into cDNA, according to the manufacturer’s instructions. RT-qPCR was then performed using an miRNA qPCR Detection kit (GeneCopoeia, Rockville, MD, USA) on an ABI 7500 thermocycler (Applied Biosystems, Carlsbad, CA, USA). The PCR conditions were set as follows: 94°C for 3 min; followed by 40 cycles of 94°C for 30 sec, 60°C for 15 sec and 72°C for 30 sec; and a final step of 82°C for 5 sec. U6 gene was used as an internal reference. The relative expression was analyzed by the 2^−ΔΔCt^ method.

### Western blotting

Tissues or cells were solubilized in cold radioimmunoprecipitation assay lysis buffer. Proteins were separated with 12% SDS-PAGE and transferred onto a polyvinylidene difluoride (PVDF) membrane, which was then incubated with Tris-buffered saline and Tween 20 (Sigma-Aldrich, St. Louis, MO, USA) containing 5% milk at room temperature for 3 h. The PVDF membrane was subsequently incubated with rabbit monoclonal anti-S1PR1 (ab125074; 1:200 dilution) and -GAPDH (ab181602; 1:200 dilution) primary antibodies (Abcam, Cambridge, UK) at room temperature for 3 h. Following washing three times with phosphate-buffered saline-Tween 20, the membrane was incubated with the goat anti-rabbit secondary antibodies (Abcam) at room temperature for 40 min. Chemiluminescent detection was performed using an enhanced chemiluminescence kit (Pierce Chemical). The relative protein expression was analyzed by Image-Pro^®^ Plus software 6.0 (Media Cybernetics, Inc., Silver Spring, MD, USA), and presented as the density ratio versus GAPDH.

### Transfection

Transfection was performed using Lipofectamine^®^ 2000 (Invitrogen Life Technologies), in accordance with the manufacturer’s instructions. For miR-148a functional analysis, the HepG2 cells were transfected with the scrambled miRNA as a negative control, miR-148a mimics or miR-148a inhibitor (Invitrogen Life Technologies). For S1PR1 functional analysis, the HepG2 cells were transfected with S1PR1-specific small interfering (si)RNA or pcDNA3.1-S1PR1 plasmid (Nlunbio, Changsha, China).

### Dual-luciferase reporter assay

A QuikChange^®^ Site-Directed Mutagenesis kit (Stratagene, La Jolla, CA, USA) was used to generate a mutant-type 3-UTR of S1PR1, according to the manufacturer’s instructions. The wild- or mutant-type 3-UTR of S1PR1 was inserted into the psiCHECK™-2 vector (Promega Corp., Madison, WI, USA). Once HepG2 cells were cultured to ~70% confluence, they were transfected with psiCHECK™-2-S1PR1-3-UTR or psiCHECK™-2-mutant S1PR1-3-UTR vector, with or without 100 nM miR-148a mimics. Following transfection for 48 h, the luciferase activities were determined on an LD400 luminometer (Beckman Coulter, Fullerton, CA, USA). Renilla luciferase activity was normalized to firefly luciferase activity.

### Invasion assay

A cell suspension containing 5×10^5^ cells/ml was prepared in serum-free media; 300 μl was added into the upper chamber and 500 μl RPMI-1640 containing 10% FBS was added into the lower chamber. Following incubation for 24 h, non-invading cells as well as the matrix gel (BD Biosciences, Franklin Lakes, NJ, USA) on the interior of the inserts was removed using a cotton-tipped swab. Invasive cells on the lower surface of the membrane were stained with crystal violet (Sigma-Aldrich) for 20 min, rinsed with water and dried in air. Five fields were randomly selected and the cell number was counted under an inverted microscope (IX83; Olympus Corporation, Tokyo, Japan) (magnification, ×100).

### Statistical analysis

The results are expressed as the mean ± standard deviation of three independent experiments. Statistical analysis of differences was performed by one-way analysis of variance using SPSS software (version 17; SPSS Inc., Chicago, IL, USA). P<0.05 was considered to indicate a statistically significant difference.

## Results

### miR-148a is downregulated and S1PR1 is upregulated in hepatocellular carcinoma tissues and cells

**Figure 1:**
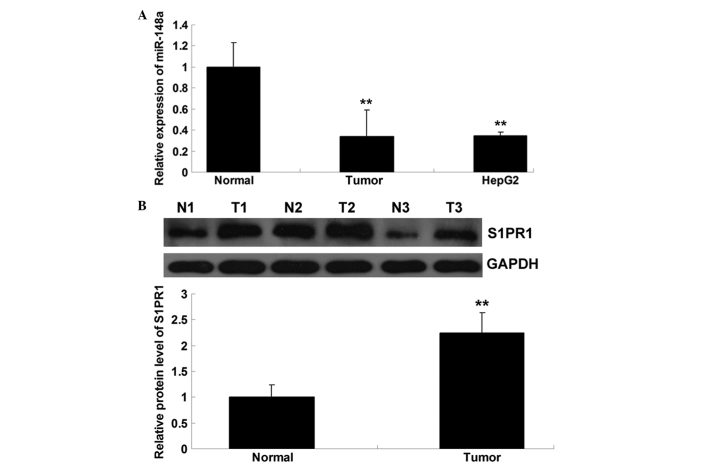
(A) Reverse transcription-quantitative polymerase chain reaction was performed to examine the relative expression of miR-148a in 20 hepatocellular carcinoma tissues (the tumor group) and that in their matched normal adjacent tissues (the normal group) and hepatocellular carcinoma HepG2 cells. (B) Western blotting was performed to determine the relative protein expression of S1PR1 in 20 hepatocellular carcinoma tissues as well as that in their matched normal adjacent tissues. The results are expressed as the mean ± standard deviation of three independent experiments. ^**^P<0.01 vs. normal. S1PR1, sphingosine-1-phosphate receptor 1; miR-148a, microRNA-148a.

Firstly, the expression level of miR-148a in hepatocellular carcinoma tissues, their matched adjacent normal tissues and hepatocellular carcinoma HepG2 cells was examined. As shown in Fig. 1A, the expression level of miR-148a in the hepatocellular carcinoma tissues was significantly reduced compared with that in the normal tissues. Consistently, the expression of miR-148a was downregulated in the hepatocellular carcinoma HepG2 cells. The protein expression of S1PR1 was determined by performing western blotting, which showed that the protein level of S1PR1 was upregulated in the hepatocellular carcinoma tissues compared with that in their matched normal adjacent tissues (Fig. 1B). Accordingly, the present data suggest that miR-148a is downregulated whereas S1PR1 is upregulated in hepatocellular carcinoma.

### S1PR1 is a direct target of miR-148a

**Figure 2:**
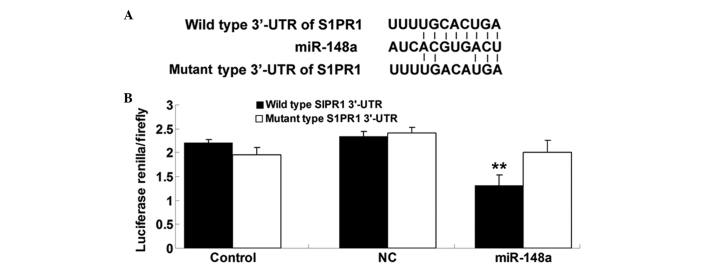
(A) Seed sequences of miR-148a in the wild- and mutant-type 3′-UTR of S1PR1. (B) Luciferase reporter assay data showed that co-transfection of HepG2 cells with miR-148a and wild-type S1PR1 3′-UTR led to a marked decrease in luciferase activity; however, co-transfection with miR-148a and mutant S1PR1 3′-UTR had no effect on luciferase activity, and co-transfection with NC miRNA and wild-type S1PR1 3′-UTR or mutant S1PR1 3′-UTR also showed no difference. ^**^P<0.01 vs. control. miR-148a, microRNA-148a; S1PR1, sphingosine-1-phosphate receptor 1; UTR, untranslated region; NC, negative control; control, cells co-transfected with blank vector and wild-type S1PR1 3′-UTR or mutant S1PR1 3′-UTR.

Bioinformatical predication was performed using TargetScan online software (http://www.targetscan.org/) and the findings showed that the putative seed sequences for miR-148a at the 3′-UTR of S1PR1 were highly conserved (Fig. 2A). To verify whether S1PR1 was a direct target of miR-148a, the wild and mutant types of S1PR1 3′-UTR were generated. The dual-luciferase reporter assay was subsequently performed in hepatocellular carcinoma HepG2 cells. As shown in Fig. 2B, the luciferase activity was significantly reduced in HepG2 cells co-transfected with the wild-type 3′-UTR of S1PR1 and miR-148a mimics, but unchanged in HepG2 cells co-transfected with the mutant S1PR1 3 UTR and miR-148a mimics, indicating that miR-148a directly binds to the 3′-UTR of S1PR1 in HepG2 cells.

### S1PR1 expression is negatively regulated by miR-148a at a transcriptional level in hepatocellular carcinoma cells

**Figure 3:**
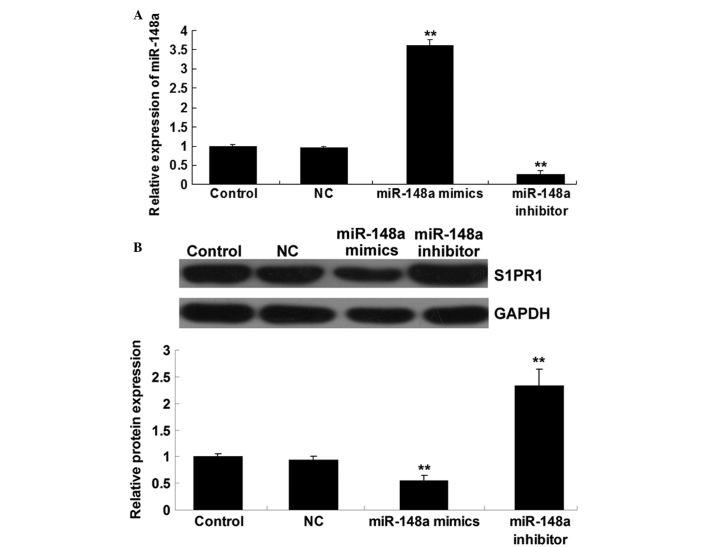
(A) Reverse transcription-quantitative polymerase chain reaction was performed to determine the relative expression of miR-148a in HepG2 cells transfected with scrambled miRNA (NC), miR-148a mimics and an miR-148a inhibitor. (B) Western blotting was performed to examine the protein level of S1PR1 in HepG2 cells transfected with scrambled miRNA (NC), miR-148a mimics and an miR-148a inhibitor. ^**^P<0.01 vs. control. miR-148a, microRNA-148a; NC, negative control; control, HepG2 cells without any transfection; S1PR1, sphingosine-1-phosphate receptor 1; miRNA, microRNA.

To further investigate the role of miR-148a in the regulation of S1PR1 expression in hepatocellular carcinoma cells, HepG2 cells were transfected with scrambled miRNA, miR-148a mimics and miR-148a inhibitor, respectively. The transfection efficiency was satisfactory (Fig. 3A). The findings showed that the protein level of S1PR1 was significantly reduced following the upregulation of miR-148a but was increased in HepG2 cells transfected with miR-148a inhibitor (Fig. 3B). These findings indicate that miR-148a plays a negative role in the regulation of S1PR1 expression at a post-transcriptional level in hepatocellular carcinoma cells.

### S1PR1 acts as a downstream effector in the miR-148a-induced inhibition of hepatocellular carcinoma cell invasion

**Figure 4:**
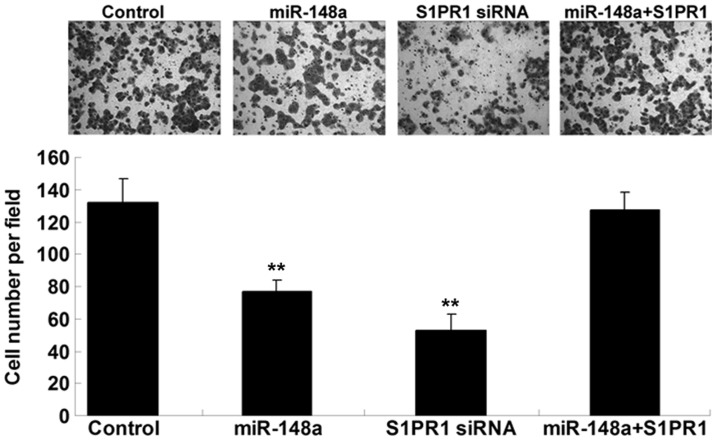
A Transwell^®^ assay was performed to determine the invasive capacity of HepG2 cells transfected with miR-148a mimics, S1PR1 siRNA, or co-transfected with miR-148a mimics and S1PR1 plasmid. ^**^P<0.01 vs. control. Control, HepG2 cells without any transfection; S1PR1, sphingosine-1-phosphate receptor 1; miR-148a, microRNA-148a; siRNA, small interfering RNA. Magnification, ×100; with crystal violet staining.

The inhibition of S1PR1 expression in HepG2 cells was used to determine whether miR-148a played a suppressive role in hepatocellular carcinoma cell invasion. HepG2 cells were transfected with miR-148a mimics or S1PR1-specific siRNA or co-transfected with miR-148a mimics and S1PR1 plasmid. A cell invasion assay was then performed. As shown in Fig. 4, the upregulation of miR-148a and inhibition of S1PR1 notably inhibited HepG2 cell invasion. In addition, the suppressive effect of miR-148a upregulation on cell invasion was reversed by S1PR1 overexpression. Based on these findings we suggest that S1PR1 acts as a downstream effector in the miR-148a-induced inhibition of hepatocellular carcinoma cell invasion.

## Discussion

In the present study, the expression level of miR-148a was shown to be notably reduced in hepatocellular carcinoma tissues and cells compared with that in normal tissues; however, the protein expression of S1PR1 was markedly upregulated. Further investigation identified S1PR1 as a direct target of miR-148a, and the protein expression of S1PR1 was negatively regulated by miR-148a in hepatocellular carcinoma cells. In addition, the current study suggested that the suppressive effect of miR-148a on hepatocellular carcinoma cell invasion is, at least partly, via the inhibition of S1PR1.

It has been well established that miRNAs can regulate various biological processes by modulating the expression of their targets at a post-transcriptional level **(**15**)**. In addition, deregulation of miRNAs has been demonstrated to be associated with the development and progression of various human malignancies **(**16**)**. In the present study, the expression level of miR-148a was shown to be notably reduced in hepatocellular carcinoma tissues and cells compared with that in normal tissues. These findings were consistent with others (17). Magrelli *et al* (17) performed a microarray and RT-qPCR to examine the miRNA expression in hepatoblastoma tissues. It was found that miR-148a was the only downregulated miRNA in hepatoblastoma tissues (17). Following the study by Magrelli *et al* (17), Yuan *et al* (18) suggested that miR-148a may be associated with hepatitis-B-virus-associated hepatocellular carcinoma. According to these and the current findings, we suggest that deregulation of miR-148a is involved in the development and progression of hepatocellular carcinoma; however, the detailed role of miR-148a in hepatocellular carcinoma, particularly the molecular regulatory mechanism, remains to be fully elucidated.

Recently, Gailhouste *et al* (14) showed that miR-148a could promote the hepatospecific phenotype, and acted as a tumor suppressor by targeting the c-Met oncogene. It was found that overexpression of miR-148a led to a notable inhibition of the invasive properties of hepatocellular carcinoma cells, whereas silencing of miR-148a promoted hepatocellular carcinoma cell invasion (14). In the present study, it was also found that miR-148a upregulation had an inhibitory effect on hepatocellular carcinoma HepG2 cell invasion. Furthermore, it was shown that S1PR1 was an important downstream effector of the miR-148a-induced inhibition of cell invasion in hepatocellular carcinoma HepG2 cells. Gailhouste *et al* (14) demonstrated that miR-148a exerted its tumor-suppressive effect by directly targeting the c-Met oncogene.

Bioactive sphingolipids, including sphingosine kinases (SKs) and their product S1P, have been demonstrated to be involved in the regulation of cancer growth, metastasis and drug resistance (19). It has been well established that S1P exerts its intracellular and extracellular pro-survival and drug resistance functions through S1PR1 (20,21); therefore, SK/S1P/S1PR1 signaling appears to be a promising therapeutic target for cancer. In the present study, it was shown that S1PR1 was significantly upregulated in hepatocellular carcinoma tissues, consistent with a previous study (9). The role of S1PR1 in the regulation of cancer cell invasion has also been previously demonstrated. For instance, S1P/S1PR1 signaling has been shown to promote cell migration and invasion via the activation of stat3 in prostate cancer cells (22). In addition, S1P/S1P1 signaling has been found to mediate Wilms tumor cell migration and invasion (23). In the present study, S1PR1 was found to be involved in the miR-148a-mediated inhibition of hepatocellular carcinoma invasion. Based on previous and the current findings, we suggest that S1PR1 could become an effective target for the prevention of hepatocellular carcinoma metastasis.

In conclusion, the present study identified S1PR1 as a direct target of miR-148a in hepatocellular carcinoma cells, and suggested that miR-148a plays a suppressive role in the regulation of hepatocellular carcinoma cell invasion, at least partially through the direct downregulation of S1PR1 expression. miR-148a may, therefore, serve as a potential therapeutic agent for hepatocellular carcinoma.
